# Association Between Racial Wealth Inequities and Racial Disparities in Longevity Among US Adults and Role of Reparations Payments, 1992 to 2018

**DOI:** 10.1001/jamanetworkopen.2022.40519

**Published:** 2022-11-07

**Authors:** Kathryn E. W. Himmelstein, Jourdyn A. Lawrence, Jaquelyn L. Jahn, Joniqua N. Ceasar, Michelle Morse, Mary T. Bassett, Bram P. Wispelwey, William A. Darity, Atheendar S. Venkataramani

**Affiliations:** 1 Division of Infectious Diseases, Department of Medicine, Massachusetts General Hospital, Harvard Medical School, Boston; 2 Department of Epidemiology and Biostatistics, Dornsife School of Public Health, Drexel University, Philadelphia, Pennsylvania; 3 François-Xavier Bagnoud Center for Health and Human Rights, Harvard T. H. Chan School of Public Health, Boston, Massachusetts; 4 The Ubuntu Center on Racism, Global Movements, and Population Health Equity, Dornsife School of Public Health, Drexel University, Philadelphia, Pennsylvania; 5 Department of Medicine, Johns Hopkins Hospital, Johns Hopkins University School of Medicine, Baltimore, Maryland; 6 Department of Pediatrics, Johns Hopkins Hospital, Johns Hopkins University School of Medicine, Baltimore, Maryland; 7 Center for Health Equity and Community Wellness, New York City Department of Health and Mental Hygiene, New York, New York; 8 Division of Global Health Equity, Department of Medicine, Brigham and Women’s Hospital, Harvard Medical School, Boston, Massachusetts; 9 New York State Department of Health, Albany; 10 Sanford School of Public Policy, Duke University, Durham, North Carolina; 11 Leonard Davis Institute of Health Economics, Division of Health Policy, Perelman School of Medicine at the University of Pennsylvania, Philadelphia

## Abstract

**Importance:**

In the US, Black individuals die younger than White individuals and have less household wealth, a legacy of slavery, ongoing discrimination, and discriminatory public policies. The role of wealth inequality in mediating racial health inequities is unclear.

**Objective:**

To assess the contribution of wealth inequities to the longevity gap that exists between Black and White individuals in the US and to model the potential effects of reparations payments on this gap.

**Design, Setting, and Participants:**

This cohort study analyzed the association between wealth and survival among participants in the Health and Retirement Study, a nationally representative panel study of community-dwelling noninstitutionalized US adults 50 years or older that assessed data collected from April 1992 to July 2019. Participants included 7339 non-Hispanic Black (hereinafter Black) and 26 162 non-Hispanic White (hereinafter White) respondents. Data were analyzed from January 1 to September 17, 2022.

**Exposures:**

Household wealth, the sum of all assets (including real estate, vehicles, and investments), minus the value of debts.

**Main Outcomes and Measures:**

The primary outcome was all-cause mortality by the end of survey follow-up in 2018. Using parametric survival models, the associations among household wealth, race, and survival were evaluated, adjusting for age, sex, number of household members, and marital status. Additional models controlled for educational level and income. The survival effects of eliminating the current mean wealth gap with reparations payments ($828 055 per household) were simulated.

**Results:**

Of the 33 501 individuals in the sample, a weighted 50.1% were women, and weighted mean (SD) age at study entry was 59.3 (11.1) years. Black participants’ median life expectancy was 77.5 (95% CI, 77.0-78.2) years, 4 years shorter than the median life expectancy for White participants (81.5 [95% CI, 81.2-81.8] years). Adjusting for demographic variables, Black participants had a hazard ratio for death of 1.26 (95% CI, 1.18-1.34) compared with White participants. After adjusting for differences in wealth, survival did not differ significantly by race (hazard ratio, 1.00 [95% CI, 0.92-1.08]). In simulations, reparations to close the mean racial wealth gap were associated with reductions in the longevity gap by 65.0% to 102.5%.

**Conclusions and Relevance:**

The findings of this cohort study suggest that differences in wealth are associated with the longevity gap that exists between Black and White individuals in the US. Reparations payments to eliminate the racial wealth gap might substantially narrow racial inequities in mortality.

## Key Points

**Question:**

What share of the longevity gap that exists between Black and White individuals in the US is explained by differences in wealth, and to what extent might reparations payments to Black individuals close this gap?

**Findings:**

In this cohort study of 7339 Black and 26 162 White middle-aged US adults, differences in survival were largely mediated by differences in household wealth. Simulations suggested that reparations payments aimed at closing the wealth gap between Black and White individuals could markedly reduce racial inequities in longevity.

**Meaning:**

These findings suggest that reparations payments may be an effective strategy to reduce racial inequities in longevity.

## Teaser

This cohort study examines the extent to which differences in wealth among Black and White individuals in the US explained observed racial disparities in survival and how reparations payments might affect racial mortality.

## Introduction

In the US, Black individuals have a higher mortality rate than White individuals,^1^ a difference that reflects the pervasive effects of structural racism. Indeed, some scholars view “group-differentiated vulnerability to premature death” as a definition of racism^2^ and suggest that inequities in excess deaths should serve as a metric of progress in eliminating racism.^3^

Racial health inequities in the US are shaped by structurally driven differential access to economic resources.^4^ Although the role of income in mediating racial health inequities is well-documented, differences in income understate the extent of overall economic inequality. Although the median income of White familes is 1.65-fold higher than that of Black families, the difference in wealth (the value of all assets owned by a household) is 8-fold.^5,6^ Because wealth is transferred intergenerationally, racial wealth gaps better reflect sustained economic disadvantages than do gaps in income or other measures.^7^ However, because wealth data are less commonly collected in surveys, few studies have examined the role of wealth in racial health inequality.^8,9,10,11^

Current racial wealth inequities arose from centuries of government policies, including 250 years of slavery, rescission of the 1865 Field Order 15 (the promise of “40 acres and a mule” to formerly enslaved people), Black codes and Jim Crow laws, official tolerance of and participation in vigilante violence, exclusion of most Black workers from New Deal programs such as Social Security, discriminatory application of the homebuying provisions of the GI Bill, government-sponsored redlining that excluded most Black families from federally guaranteed mortgages, discriminatory criminal punishment, and lax enforcement of Civil Rights era laws designed to end discrimination in employment, policing, housing, health care, and lending.^12,13,14^ The ideology of “colorblindness” has helped to perpetuate the effects of these policies into the present.^15^

Reparations payments to Black individuals have been proposed to redress these injustices and, consequently, narrow racial wealth and health gaps.^3,16,17^ Such payments would be in keeping with past instances of reparations, such as the US government’s reparations to Japanese Americans interned during WWII and the German government’s payments to Holocaust survivors under the Luxembourg Agreement.^18^

To assess the role of wealth in mediating racial longevity gaps—and whether narrowing wealth differences with reparations payments might mitigate racial mortality differences—we analyzed longitudinal data on middle-aged and older adults from the Health and Retirement Study (HRS), which collects information on both mortality and wealth.^19^ We first examined the extent to which racial differences in wealth explained observed racial inequities in survival. We then modeled how reparations payments might affect racial mortality differences.

## Methods

The University of Pennsylvania does not require institutional review board approval or informed consent for analysis of deidentified, publicly available data.^19^ This cohort study follows the Strengthening Reporting of Observational Studies in Epidemiology (STROBE) reporting guideline.

### Data and Study Population

The HRS, sponsored by the National Institute on Aging and conducted by the University of Michigan, is a nationally representative longitudinal study of community-dwelling noninstitutionalized US adults 50 years or older. The HRS has collected demographic, economic, and health data since 1992.^20^ Participants, who enter the study at different times, are surveyed every 2 years in waves until death or loss to follow-up.

The RAND HRS Longitudinal File 2018 (V1),^20^ which we analyzed, includes data on 42 233 respondents. Our sample included respondents from all waves of data collection between April 1992 and July 2019 (the most recent year for which data are available). Although the HRS also collects information on respondents’ spouses, we excluded spouses owing to higher rates of missing data and loss to follow-up. Because our analysis focused on wealth and health gaps between Black and White US residents, we included only US-born respondents self-identifying as non-Hispanic/non-Latino Black or non-Hispanic/non-Latino White. Our final sample included 33 501 (7339 Black and 26 162 White) participants from all HRS waves between 1992 and 2019. Although some participants missed some waves of data collection, values for all demographic variables were drawn from the earliest wave of study participation, minimizing missing data.

### Wealth and Income Measures

The HRS calculates wealth at the household level as the sum of all assets minus the value of debts. Assets queried include the net value of primary and secondary residences; other real estate; vehicles; businesses; individual retirement and Keogh accounts; stocks; mutual funds; investment trusts; checking, savings, and money market accounts; certificates of deposit; government savings bonds; treasury bills; bonds; bond funds; and other savings. Debts queried include mortgages and land contracts on residences, home loans, and other debt.

For each wealth component, respondents are asked whether they own the given asset and, if so, its estimated value. If the respondent does not know the value, the interviewer uses an “unfolding brackets” technique, asking a series of questions about the value of the asset that becomes more specific based on the respondent’s answers. The HRS uses these answers and data from other study waves to impute the value of assets when necessary. Since 2002, the HRS has asked respondents to confirm or correct the value of wealth components.

Because costs associated with illness (eg, medical bills, lost wages) might depress income and wealth over time while also contributing to early mortality, we used figures for household income and wealth at study entry. Due to the highly skewed distribution of wealth, we analyzed wealth using deciles. All dollar figures were adjusted to 2018 US dollars using the Consumer Price Index.

### Mortality

Our main outcome was survival conditional on entry into the HRS. The HRS derives the month and year of death from postmortem interviews with family members and the National Death Index.

### Reparations Payments

Reparations advocates have suggested different figures for the appropriate amount of reparations payments. Marketti^21^ estimated the value of wages denied to enslaved workers at $2.1 to $4.7 trillion in 1983, approximately $211 000 to $473 000 per Black individual in current dollars.^22^ Craemer et al^12^ have suggested figures as high as $300 million per person, based on the value of lost freedom, wages, and assets, with compound interest. Darity and Mullen^23^ have advocated closing the mean pretax wealth gap via payments of approximately $800 000 per Black household.

Most scholars have benchmarked their estimates to the amount required to equalize the per-household wealth of Black and White US residents.^14,24^ Hence, we modeled the longevity effects of reparations payments equivalent to the mean 2019 household wealth gap of $840 900 ($828 055 in 2018 US dollars).^25^

### Statistical Analysis

We examined the association between longevity and race using parametric Weibull survival models. All models were adjusted for self-reported race. Initial models included adjustment for age, sex, marital status, and number of household members. In the HRS, sex data are derived primarily from self-report.

To examine the extent to which wealth explained associations between race and survival, we then adjusted for wealth deciles. Studies of intergenerational wealth transfers suggest that household wealth is a major driver of offspring’s incomes and educational achievement, and that income and educational attainment may therefore mediate the effects of wealth on health. Hence, our primary analyses modeled the effects of reparations without adjusting for income and educational attainment (ie, assuming that reparations would, at least in the long term, attenuate observed racial differences in educational attainment and income).^7,26^ However, we conducted sensitivity analyses that also adjusted for income deciles and level of educational attainment. The estimates from these sensitivity analyses may represent a lower bound, because they impose the likely inaccurate assumption that wealth does not influence income or educational attainment.

To account for the nonlinear association between age and survival and for differential timing of entry into the HRS, we adjusted for age at study entry and age at study entry squared in our Weibull survival model. We also conducted a sensitivity analysis using semiparametric Cox proportional hazards regression models that, beyond adjusting for age at baseline, explicitly accounted for left truncation (ie, the fact that individuals who died before reaching the age of study entry were not included in the sample).^27^ Because Cox models generate proportional hazards (rather than absolute risks or hazards), outputs from these models cannot be readily used for predictive modeling, such as our simulation of reparations payments. For this reason, our primary analysis relied on parametric Weibull survival models.

We used the coefficients from our survival models to estimate survival for Black participants after adding reparations payments of $828 055 (in 2018 dollars) to their household’s wealth at study entry.^28,29^ We calculated the difference in median survival between Black and White participants after reparations, expressed as a percentage change from the difference without reparations. We conducted this exercise using 2 sets of models. In the first, we adjusted for demographic characteristics, educational attainment, and household income; in the second, we adjusted only for demographic characteristics and did not include educational attainment or household income.

Owing to small numbers of Black participants in upper wealth deciles, using Black individual–specific regression coefficients to derive our projections generated wide 95% CIs. Therefore, we used regression coefficients derived from the entire study cohort in our principal analysis, a strategy that incorporated the disputed assumption that Black and White individuals experience the same health benefits from a given level of wealth.^30^ In sensitivity analyses, we conducted simulations using the subsample of Black participants only.

Analyses were performed with SAS, version 9.4 (SAS Institute Inc),^31^ and RStudio, version 2022.02.3 + 492 (R Project for Statistical Computing),^32^ using survey procedures that account for the complex sample design and HRS-provided weights that yield national estimates. Two-sided *P* < .05 indicated statistical significance. Data analysis was conducted between January 1 and September 17, 2022.

## Results

Our final sample consisted of 33 051 participants (14 706 men [44.5%] and 18 795 [56.9%]; mean [SD] age, 59.3 [11.1] years); their characteristics and weighted percentages are displayed in Table 1. A total of 7339 participants were Black and 26 162 were White. Compared with White participants, Black participants were younger (mean [SD] age, 58.2 [6.1] vs 59.6 [6.8] years), more often female (4383 [54.7%] vs 14 412 [49.7%]), and more likely to be unmarried (4063 [52.0%] vs 9402 [30.2%]) and had lower levels of educational attainment (bachelor’s degree, 600 [9.5%] vs 3568 [16.6%]). Black participants’ median life expectancy was 77.5 (95% CI, 77.0-78.2) years, 4 years shorter than the median life expectancy for White participants (81.5 [95% CI, 81.2-81.8] years).

**Table 1.** Characteristics of Health and Retirement Study Participants, 1992 to 2018

	Participant group^a^	
	Black (n = 7339)	White (n = 26 162)
Age at study entry, mean (SD), y	58.2 (6.1)	59.6 (6.8)
Sex		
Men	2956 (45.3)	11 750 (50.3)
Women	4383 (54.7)	14 412 (49.7)
Marital status		
Married or partnered at study entry	3276 (48.0)	16 760 (69.8)
Unmarried	4063 (50.2)	9402 (30.2)
Educational level		
Less than high school diploma	2413 (30.0)	4873 (14.7)
GED or high school diploma	3566 (49.8)	14 205 (52.9)
Some college	432 (6.0)	1359 (6.3)
Bachelor’s degree	600 (9.5)	3568 (16.6)
Graduate degree	328 (4.7)	2157 (9.5)
No. of people in household, mean (SE)	2.5 (0.02)	2.4 (0.01)
Income decile (range)		
1 (<$12 034)	1445 (20.6)	1582 (6.0)
2 ($12 034-$18 974)	1001 (12.8)	2049 (5.0)
3 ($18 975-$26 956)	781 (10.1)	2373 (7.8)
4 ($26 957-$35 913)	708 (8.6)	2519 (8.2)
5 ($35 914-$46 492)	633 (9.2)	2645 (9.1)
6 ($46 493-$59 831)	635 (8.6)	2557 (8.9)
7 ($59 832-$77 795)	622 (8.6)	2806 (10.7)
8 ($77 796-$104 632)	552 (7.9)	2943 (11.9)
9 ($104 633-$156 934)	549 (7.5)	3224 (14.2)
10 (≥$156 935)	413 (6.1)	2464 (16.1)
Wealth decile (range)		
1 (<$1234)	1539 (22.7)	1769 (7.3)
2 ($1234-$24 437)	1465 (21.0)	1949 (7.4)
3 ($24 438-$65 006)	1119 (15.5)	2208 (9.1)
4 ($65 007-$116 068)	870 (12.1)	2461 (10.0)
5 ($116 069-$182 538)	657 (8.6)	2630 (10.2)
6 ($182 539-$273 594)	524 (7.2)	2626 (10.4)
7 ($273 595-$409 042)	374 (5.3)	2811 (11.4)
8 ($409 043-$635 181)	240 (3.1)	2806 (11.1)
9 ($635 182-$1 861 188)	181 (3.0)	2840 (11.2)
10 (≥$1 861 189)	88 (1.5)	2840 (12.0)
Missing	282	1222

Abbreviation: GED, General Educational Development.

^a^ Unless otherwise indicated, data are expressed as No. (%) of participants. All numerical values are unweighted; all means and percentages are weighted. Percentages are rounded and may not total 100. Income and wealth deciles were generated using the entire study population.

The household incomes of Black participants were lower; 1445 (20.6%) of Black participants fell in the lowest income decile, and only 413 (6.1%) in the top decile. In contrast, only 1582 White participants (6.0%) had incomes in the lowest decile, and 2464 (16.1%) had incomes in the top decile. Consistent with prior studies,^11^ we found even larger racial differences in wealth; 1539 Black participants (22.7%) vs 1769 White participants (7.3%) lived in households in the lowest decile of wealth, and 88 Black participants (1.5%) vs 2840 White participants (12.0%) lived in households in the top wealth decile. The median wealth of Black households was $31 500 (IQR, 0-$130 050), compared with $215 785 (IQR, $64 410-$536 105) for White households.

Table 2 reports results from the Weibull survival models, adjusted for (1) demographic variables only, (2) income, (3) educational attainment, (4) household wealth, and (5) wealth, income, and educational attainment. In all models, being male (hazard ratio [HR] adjusted for wealth, income, and educational attainment, 1.60 [95% CI, 1.54-1.66]) was associated with greater hazards of death, whereas being married or partnered (HR adjusted for wealth, income, and educational attainment, 0.94 [95% CI, 0.89-1.00]) was associated with lower hazards of death. Models without adjustment for wealth, income, or educational attainment revealed a significant racial gap in survival, with Black participants having an HR of death of 1.26 (95% CI, 1.18-1.34) compared with White participants.

**Table 2.** Hazard Ratios (HRs) for Mortality Among Middle-aged US Adults Derived From Weibull Models, 1992 to 2018

Variable	Model adjustment, HR (95% CI)^a^				
	No SES variables	Income	Educational attainment	Wealth	Wealth, income, and educational attainment
Age, y					
At study entry	0.99 (0.99-1.00)	0.99 (0.99-0.99)	0.99 (0.99-1.00)	0.99 (0.99-1.00)	0.99 (0.99-0.99)
At study entry squared	1.00 (1.00-1.00)	1.00 (1.00-1.00)	1.00 (1.00-1.00)	1.00 (1.00-1.00)	1.00 (1.00-1.00)
Men (vs women)	1.54 (1.49-1.60)	1.62 (1.57-1.69)	1.57 (1.51-1.63)	1.55 (1.49-1.61)	1.60 (1.54-1.66)
Married or partnered (vs unpartnered)	0.71 (0.68-0.75)	0.92 (0.87-0.96)	0.73 (0.70-0.76)	0.87 (0.82-0.91)	0.94 (0.89-1.00)
No. of household residents	1.02 (0.99-1.04)	1.01 (0.99-1.03)	1.01 (0.98-1.03)	0.99 (0.97-1.02)	0.99 (0.97-1.02)
Black (vs White)	1.26 (1.18-1.34)	1.09 (1.02-1.16)	1.12 (1.05-1.20)	1.00 (0.92-1.08)	0.95 (0.87-1.03)
Wealth decile (range) (vs first decile, <$1234)					
2 ($1234-$24 437)	NA	NA	NA	0.78 (0.70-0.87)	0.82 (0.74-0.91)
3 ($24 438-$65 006)	NA	NA	NA	0.66 (0.60-0.73)	0.72 (0.65-0.79)
4 ($65 007-$116 068)	NA	NA	NA	0.61 (0.55-0.68)	0.69 (0.62-0.76)
5 ($116 069-$182 538)	NA	NA	NA	0.54 (0.49-0.60)	0.62 (0.55-0.69)
6 ($182 539-$273 594)	NA	NA	NA	0.53 (0.48-0.58)	0.63 (0.57-0.71)
7 ($273 595-$409 042)	NA	NA	NA	0.48 (0.43-0.54)	0.59 (0.52-0.67)
8 ($409 043-$635 181)	NA	NA	NA	0.44 (0.40-0.49)	0.57 (0.51-0.64)
9 ($635 182-$1 861 188)	NA	NA	NA	0.39 (0.36-0.43)	0.54 (0.49-0.60)
10 (≥$1 861 189)	NA	NA	NA	0.39 (0.25-0.44)	0.59 (0.52-0.68)
Income decile (range) (vs first decile, <$12 034)					
2 ($12 034-$18 974)	NA	0.82 (0.76-0.89)	NA	NA	0.85 (0.78-0.92)
3 ($18 975-$26 956)	NA	0.73 (0.66-0.81)	NA	NA	0.81 (0.73-0.89)
4 ($26 957-$35 913)	NA	0.66 (0.59-0.73)	NA	NA	0.77 (0.69-0.87)
5 ($35 914-$46 492)	NA	0.63 (0.57-0.70)	NA	NA	0.79 (0.71-0.87)
6 ($46 493-$59 831)	NA	0.55 (0.49-0.61)	NA	NA	0.70 (0.62-0.79)
7 ($59 832-$77 795)	NA	0.49 (0.44-0.55)	NA	NA	0.64 (0.56-0.73)
8 ($77 796-$104 632)	NA	0.47 (0.43-0.51)	NA	NA	0.64 (0.57-0.71)
9 ($104 633-$156 934)	NA	0.37 (0.33-0.42)	NA	NA	0.53 (0.46-0.60)
10 (≥$156 935)	NA	0.34 (0.31-0.38)	NA	NA	0.51 (0.44-0.58)
Educational level (vs less than high school diploma)					
GED or high school diploma	NA	NA	0.77 (0.74-0.80)	NA	0.89 (0.85-0.94)
Some college	NA	NA	0.71 (0.63-0.80)	NA	0.90 (0.78-1.04)
Bachelor’s degree	NA	NA	0.55 (0.51-0.60)	NA	0.74 (0.68-0.81)
Graduate degree	NA	NA	0.48 (0.44-0.53)	NA	0.70 (0.63-0.77)

Abbreviations: GED, General Educational Development; NA, not applicable; SES, socioeconomic status.

^a^ All values were calculated based on weighted populations. The 95% CIs were calculated using appropriate strata and cluster variables to account for the Health and Retirement Study’s complex design.

Adjustment for wealth entirely attenuated the estimated racial gap in survival with the HR for Black individuals, decreasing to 1.00 (95% CI, 0.92-1.08). In contrast, in models adjusted for income or educational attainment alone, Black participants continued to have significantly greater hazards of death (HR, 1.09 [95% CI, 1.02-1.16] when adjusting for income; HR, 1.12 [95% CI, 1.05-1.20] when adjusting for educational attainment). The hazard of death decreased with each higher wealth decile, although the largest decreases were observed for the bottom 7 deciles (eFigure 1 in the Supplement).

In the final model, which adjusted for educational attainment, wealth, and income, Black individuals’ hazard of death was not significantly different from that of White individuals (HR, 0.95 [95% CI, 0.87-1.03]). Greater wealth remained associated with survival (HR for highest decile, 0.59 [95% CI, 0.52-0.68]), although with greater HRs than in models excluding income and educational attainment (HR for highest decile, 0.39 [95% CI, 0.25-0.44]). Higher income was also associated with lower hazard of death (HR for highest decile, 0.34 [95% CI, 0.31-0.38]). Similarly, in semiparametric Cox proportional hazards regression models accounting for left truncation, adjusting for wealth (but not income or educational attainment) attenuated elevated hazard of death for Black participants (eTable 1 in the Supplement). The coefficient estimates from the Cox proportional hazards regression models were nearly identical to those from the Weibull model.

Table 3 reports the median survival of Black and White participants with and without reparations payments of $828 055 (in 2018 US dollars) to Black households (eFigure 2 in the Supplement for prereparations and postreparations wealth distributions for each racial group). These projections, derived from regression coefficients from the Weibull survival models, suggest that reparations payments might shrink the gap in median survival from 4 years to 1.4 years (in models adjusting for income and educational attainment) or even −0.1 years (ie, Black median survival might slightly exceed White survival) in models excluding income and educational attainment (ie, reduction of longevity gap by 65.0% to 102.5%). eTable 2 in the Supplement shows the same estimates derived from models constructed with Black participants only, which are similar in magnitude but with wider 95% CIs. Consistent with these findings, the Figure illustrates the simulated effects of reparations payments across the entire survival distribution, using models with and without adjustment for income and educational level.

**Table 3.** Median Life Expectancy of Black and White HRS Participants and Projected Life Expectancy of Black Participants After Reparations Payments

Outcome	Participants			
	White	Black	Black with reparations	
			Models with all SES variables	Wealth-only models
Median life expectancy (95% CI), y^a^	81.5 (81.2 to 81.8)	77.5 (77.0 to 78.2)	80.1 (79.5 to 80.7)	81.6 (81.0 to 82.2)
Difference compared with White participants, y	NA	4.0	1.4	−0.1
*P* value^b^		<.001	.002	.70
Difference in longevity gap accounted for by reparations payments, %	NA	NA	65.0	102.5

Abbreviations: HRS, Health and Retirement Study; NA, not applicable; SES, socioeconomic status.

^a^ Estimated using the Weibull models reported in Table 2, which account for the HRS’ complex sample design.

^b^ Calculated using the Wilcoxon signed-rank test.

**Figure.**
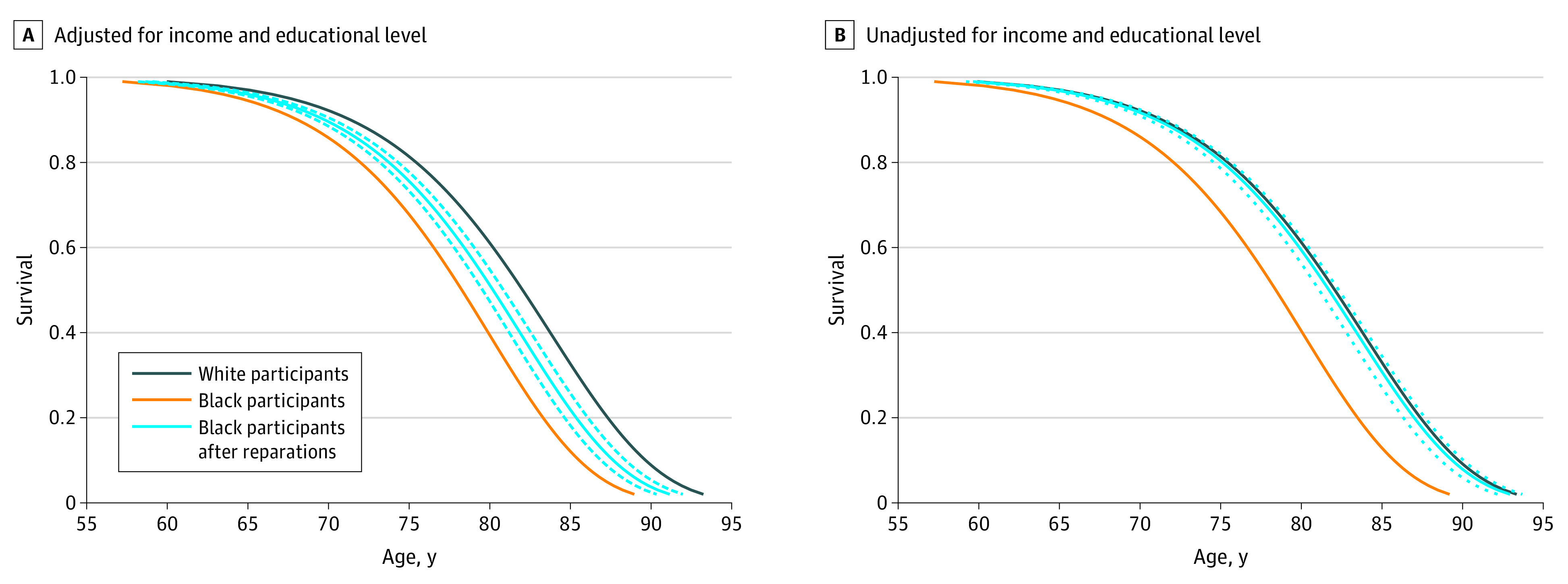
Survival of White and Black Participants and Projected Survival of Black Participants After Reparations Payments Data are from the Health and Retirement Study. Survival curves are drawn for an individual with the mean value of all covariates and using the Weibull survival models from Table 2. Dashed lines represent 95% CIs.

## Discussion

The findings of this cohort study suggest that Black middle-aged and older adults have median survival 4.0 years shorter than their White counterparts. Our analyses suggest that differences in wealth account for a considerable portion of these inequities in survival, and that closing the wealth gap with reparations payments might substantially diminish inequities in longevity among Black and White individuals.

Our findings are concordant with previous studies documenting a strong association between wealth and health.^33,34,35^ A meta-analysis of 29 studies^33^ found wealth was associated with better health, even when controlling for other measures of socioeconomic status. Another study^34^ found that wealth was associated with self-reported health. One analysis^35^ estimated that individuals with negative wealth had a 62% higher risk of death than those in the highest wealth quintile. In line with the present study, simulations by Richardson et al^16^ suggested that reparations payments to Black individuals might have reduced rates of COVID transmission in Louisiana. Studies of cash transfers in low- and middle-income countries have similarly found health benefits for both children and adults.^36^

Wealth may improve health through stable access to health care, housing, food, and education, while offering protection from chronic stress associated with economic uncertainty.^8^ Parental wealth plays a key role in children’s educational and economic opportunities.^7,26^ The benefits of reparations payments might be amplified by neighborhood-level effects; because of residential segregation, reparations payments would be concentrated in Black neighborhoods, boosting community-level resources (eg, supermarkets, well-funded public schools) that support health.^4,37^

Moreover, transferring wealth to Black US residents would likely aid Black-serving institutions such as historically Black colleges and universities and safety-net hospitals, which have fewer resources than their White-serving counterparts.^38,39^ Scholars have also theorized that reparations might have psychological benefits for recipients.^40^

However, reparations payments alone would not fully address structural determinants of health that would continue to be operative, such as political power, social capital, incarceration, policing, and discrimination in health care.^41^ They could not fully repay Black people for the harms their ancestors experienced during enslavement; loss of connections to one’s lineage, land, and indigenous knowledge cannot be restored with cash. Moreover, without eliminating ongoing practices that harm Black households, such as discriminatory lending and disproportionate burdens of penalties and fines, the benefits of one-time reparations payments would likely erode over time.^14,42^

### Limitations

The most important limitation of our study is that our findings merely document the association between wealth and survival, and any causal interpretation rests on the unproven assumption that greater wealth improves health. Although analyses of wealth shocks or other quasi-experimental variation might support a causal relationship, such analyses may fail to capture the effects of overall racial wealth inequities, which are largely inherited (eg, through the inheritance of property or educational opportunity), reflecting the long-term effects of structural racism.^7,12,14,21,23,24,42^ Because our interest was in capturing the consequences of historically mediated wealth gaps, we opted for a more associational approach. We note that our approach, by focusing on wealth at baseline, addresses (at least in part) concerns related to reverse causality (ie, that robust health facilitates asset accumulation).^43^

The HRS does not identify Indigenous individuals (eg, Native American, Alaskan Native, Native Hawaiian) who, like Black individuals, have a well-founded claim to reparations and markedly reduced longevity.^44^ The small number of Black participants in upper wealth deciles precluded using Black-specific survival models to derive our estimates of the effects of reparations, requiring the use of regression coefficients derived from our entire population. If, as some suggest, higher socioeconomic status results in fewer health benefits for Black individuals than for White individuals, the survival benefits of reparations could be lower than we project.^30^

We note that, owing to the highly unequal distribution of wealth in the US, reparations would result in most Black households having greater wealth than the median White household. We are unable in our analysis to fully explore how reparations-induced changes in the overall US wealth distribution, and concomitant sociopolitical changes, might affect the longevity of Black individuals.

Because the HRS enrolls older adults, our analysis is restricted to survival among adults 50 years or older. Black individuals who survive to 50 years of age may have unique traits that support health despite the deleterious effects of structural racism. Moreover, although more than 90% of deaths among both Black and White individuals occur after 50 years of age, mortality at younger ages is 52% higher among Black individuals.^45^ Wealth transfers to younger Black individuals might attenuate differences in early deaths, causing us to potentially underestimate the salutary effects of reparations.

The life expectancy of US residents of all races, particularly poor residents, has declined in recent years^46^; hence, the health benefits of wealth redistribution programs may extend beyond reparations, a question not explored in our study. Finally, many reparations proposals call for comprehensive redress beyond wealth transfers, including free higher education and forgiveness of student loan debts, land grants, investments in Black cultural assets, educational materials reflecting accomplishments of Black people and communities, corporate regulation, and a guaranteed living wage.^47^ The potential effects of such programs and of reparations payments of alternative amounts or dispersed over different periods represent an important avenue for further investigation.^14^

## Conclusions

Racism harms the health and well-being of Black individuals through multiple mechanisms. Our study explores the importance of one mechanism—wealth inequality—through which past and present racial injustices increase mortality. Reparations payments are a means to redress the harms of enslavement and other racist policies and practices. Our study explores the importance of one mechanism—wealth inequality—through which past and present racism is associated with increased mortality.

## Notes

Supplement. **eTable 1.** Hazards Ratios of the Effects of Wealth, Income, and Educational Attainment on Mortality for Middle-Aged US Adults Derived From Cox Proportional Hazards Models (1992-2018) **eTable 2.** Median Life Expectancy of Black and White HRS Participants and Projected Life Expectancy of Black Participants After Reparations Payments, Using Coefficients From Models Constructed Using Only Black Participants **eFigure 1.** Relationship Between Life Expectancy and Wealth Among HRS Participants (1992-2018) **eFigure 2.** Wealth Distribution for Black and White HRS Participants (1992-2018) and Simulated Wealth Distribution After Reparations Payments to Black Participants
