# Supplementary material for: Association Between Racial Wealth Inequities and Racial Disparities in Longevity Among US Adults and Role of Reparations Payments, 1992 to 2018
Source: JAMA Netw Open. 2022 Nov 7;5(11):e2240519. doi: 10.1001/jamanetworkopen.2022.40519 (PMC9641537; doi:10.1001/jamanetworkopen.2022.40519)
Supplement: Supplement. — eTable 1. Hazards Ratios of the Effects of Wealth, Income, and Educational Attainment on Mortality for Middle-Aged US Adults Derived From Cox Proportional Hazards Models (1992-2018) eTable 2. Median Life Expectancy of Black and White HRS Participants and Projected Life Expectancy of Black Participants After Reparations Payments, Using Coefficients From Models Constructed Using Only Black Participants eFigure 1. Relationship Between Life Expectancy and Wealth Among HRS Participants (1992-2018) eFigure 2. Wealth Distribution for Black and White HRS Participants (1992-2018) and Simulated Wealth Distribution After Reparations Payments to Black Participants [file jamanetwopen-e2240519-s001.pdf]

Supplementary Online Content

Himmelstein KEW, Lawrence JA, Jahn JL, et al. Association between racial wealth inequities and racial disparities in longevity among US adults and role of reparations payments, 1992 to 2018. *JAMA Netw Open*. 2022;5(11):e2240519. doi:10.1001/jamanetworkopen.2022.40519

eTable 1. Hazards Ratios of the Effects of Wealth, Income, and Educational Attainment on Mortality for Middle-Aged US Adults Derived From Cox Proportional Hazards Models (1992-2018)

eTable 2. Median Life Expectancy of Black and White HRS Participants and Projected Life Expectancy of Black Participants After Reparations Payments, Using Coefficients From Models Constructed Using Only Black Participants

eFigure 1. Relationship Between Life Expectancy and Wealth Among HRS Participants (1992-2018)

eFigure 2. Wealth Distribution for Black and White HRS Participants (1992-2018) and Simulated Wealth Distribution After Reparations Payments to Black Participants

This supplementary material has been provided by the authors to give readers additional information about their work.

© 2022 Himmelstein KEW et al. *JAMA Network Open*.

eTable 1. Hazards Ratios of the Effects of Wealth, Income, and Educational Attainment on Mortality for Middle-Aged US Adults Derived From Cox Proportional Hazards Models (1992-2018)

	**No SES variables** (*P* value)	**Income** (*P* value)	**Education** (*P* value)	**Wealth** (*P* value)	**Wealth, income, and education** (*P* value)
**Male** (vs. female)	1.54 (<0.001)	1.61 (<0.001)	1.56 (<0.001)	1.54 (<0.001)	1.57 (<0.001)
**Married or partnered** (vs. unpartnered)	0.74 (<0.001)	0.95 (0.03)	0.77 (<0.001)	0.92 (0.004)	0.99 (0.77)
**Number of household residents**	1.02 (0.15)	1.01 (0.24)	1.01 (0.39)	1.00 (0.93)	1.00 (0.75)
**Black** (vs. White)	1.26 (<0.001)	1.10 (0.003)	1.14 (<0.001)	1.02 (0.55)	0.99 (0.71)
**Wealth decile** (vs. first decile, <$1,234)					
2 ($1,234-24,437)				0.81 (<0.001)	0.83 (0.0001)
3 ($24,438-65,006)				0.69 (<0.001)	0.73 (<0.001)
4 ($65,007-116,068)				0.64 (<0.001)	0.70 (<0.001)
5 ($116,069-182,538)				0.58 (<0.001)	0.65 (<0.001)
6 ($182,859-273,594)				0.56 (<0.001)	0.65 (<0.001)
7 ($273,595-409,042)				0.51 (<0.001)	0.60 (<0.001)
8 ($409,043-635,181)				0.47 (<0.001)	0.58 (<0.001)
9 ($635,182-1,861,188)				0.43 (<0.001)	0.55 (<0.001)
10 (≥ $1,861,189)				0.42 (<0.001)	0.60 (<0.001)
**Income decile** (vs. first decile, <$12,034)					
2 ($12,034-18,974)		0.84 (<0.001)			0.86 (<0.001)
3 ($18,975-26,956)		0.76 (<0.001)			0.85 (0.001)
4 ($26,057-35,913)		0.69 (<0.001)			0.83 (0.001)
5 ($35,914-46,492)		0.67 (<0.001)			0.85 (<0.001)
6 ($46,493-59,831)		0.58 (<0.001)			0.76 (<0.001)
7 ($59,832-77,795)		0.52 (<0.001)			0.70 (<0.001)
8 ($77,796-104,632)		0.50 (<0.001)			0.70 (<0.001)
9 ($104,633-156,934)		0.40 (<0.001)			0.58 (<0.001)
10 (≥ $156,935)		0.36 (<0.001)			0.56 (<0.001)
**Education level** (vs. less than HS diploma)					
GED or HS diploma			0.79 (<0.001)		0.94 (0.03)
Some college			0.72 (<0.001)		0.94 (0.39)
Bachelor's degree			0.58 (<0.001)		0.78 (<0.001)
Graduate degree			0.51 (<0.001)		0.75 (<0.001)

Note: SES = socioeconomic status. All values are calculated based on weighted populations. The 95% CIs were calculated using appropriate strata and cluster variables to account for the Health and Retirement Study's complex design. All models account for left-truncation of the data.

© 2022 Himmelstein KEW et al. *JAMA Network Open*.

eTable 2. Median Life Expectancy of Black and White HRS Participants and Projected Life Expectancy of Black Participants After Reparations Payments, Using Coefficients From Models Constructed Using Only Black Participants

	White participants	Black HRS participants	Black HRS participants with reparations (models including all SES variables)	Black HRS participants with reparations (wealth-only models)
Median life expectancy (95% CI)	81.5 (81.2-81.8)	77.5 (77.0-78.2)	79.8 (78.2-81.4)	82.1 (80.5-83.6)
Difference in years as compared to White participants (p-value)	N/A	4 (<0.001)	1.7 (0.01)	-0.6 (0.88)
Percent difference in longevity gap accounted for by reparations payments	N/A	N/A	58.3%	114.3%

Note: HRS = Health and Retirement Study, SES = socioeconomic status, N/A = not applicable. Median life expectancy with reparations was predicted using the Weibull models constructed using only Black HRS participants, which account for the HRS' complex sample design. p-values for difference in median survival calculated using the Wilcoxon signed-rank test.

© 2022 Himmelstein KEW et al. *JAMA Network Open*.

eFigure 1. Relationship Between Life Expectancy and Wealth Among HRS Participants (1992-2018)

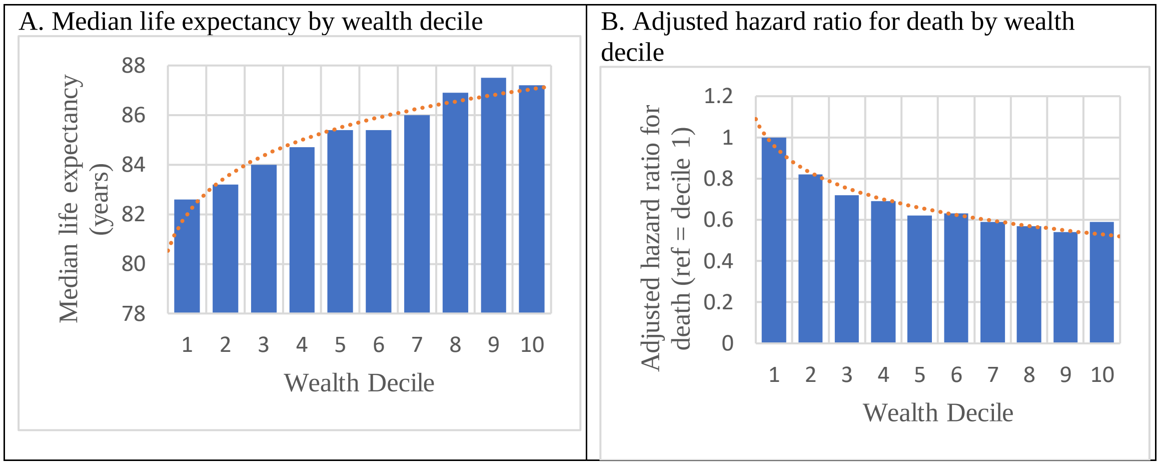

Note: HRS = Health and Retirement Study. Life expectancies in Panel A are unadjusted for covariates; hazard ratios in panel B are adjusted for age at study entry, (age at study entry)^2, gender, self-reported race, number of household residents, and marital status.

© 2022 Himmelstein KEW et al. *JAMA Network Open*.

eFigure 2. Wealth Distribution for Black and White HRS Participants (1992-2018) and Simulated Wealth Distribution After Reparations Payments to Black Participants

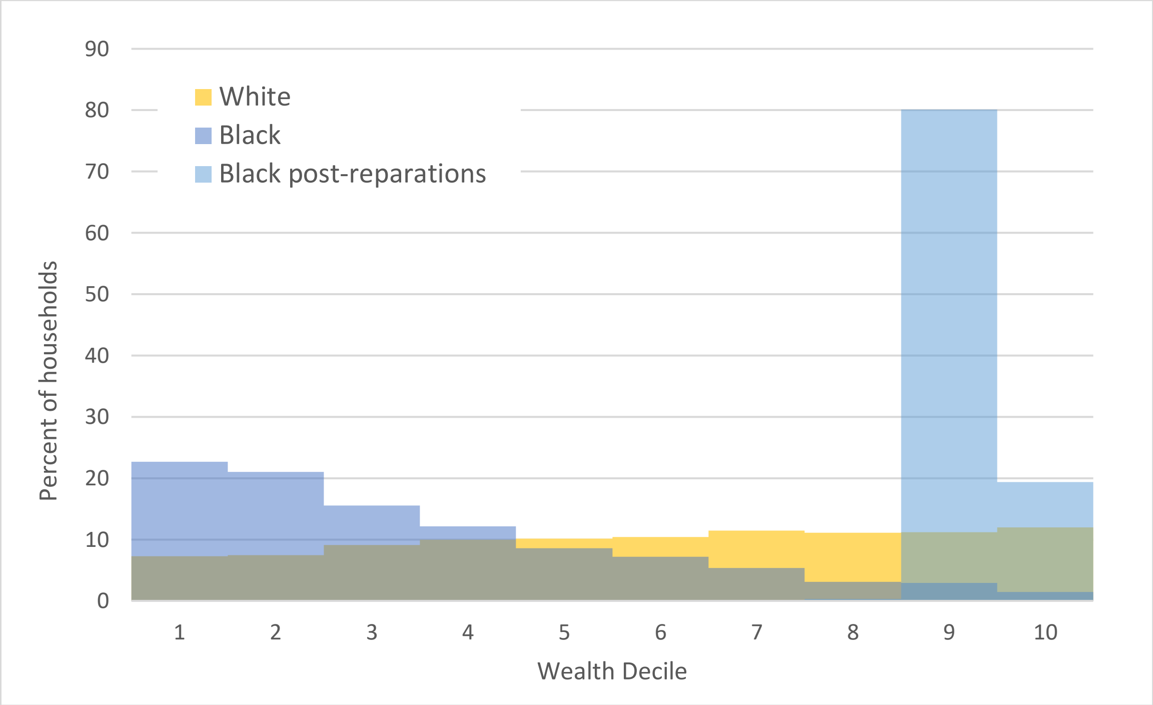

Note: HRS = Health and Retirement Study.

© 2022 Himmelstein KEW et al. *JAMA Network Open*.
